# Health-related quality of life associated with fatigue, physical activity and activity pacing in adults with chronic conditions

**DOI:** 10.1186/s13102-025-01057-x

**Published:** 2025-01-28

**Authors:** Ioulia Barakou, Bregje L. Seves, Ulric S. Abonie, Tracy Finch, Kate L. Hackett, Florentina J. Hettinga

**Affiliations:** 1 Department of Nursing, Midwifery & Health, Northumbria University, Newcastle upon Tyne, UK; 2 Department of Sport Exercise and Rehabilitation, Northumbria University, Newcastle upon Tyne, UK; 3 Department of Rehabilitation Medicine, University Medical Center Groningen, University of Groningen, Groningen, The Netherlands; 4 Department of Social Work, Education and Community Wellbeing, Northumbria University, Newcastle upon Tyne, NE7 7XA UK; 5 Department of Human Movement Sciences, Vrije Universiteit Amsterdam, Van der Boechorststraat 9, Amsterdam, 1081BT The Netherlands

**Keywords:** Exercise, Chronic disease, Fatigue, Energy regulation, Long-term conditions

## Abstract

**Background:**

Fatigue and inactivity are linked to decreased health-related quality of life (HRQoL) in chronic conditions. A multidimensional approach to activity pacing may improve HRQoL by promoting physical activity (PA) and alleviating fatigue. Addressing fatigue across chronic conditions is crucial, especially when underlying causes are unknown. This study aimed to (1) examine associations between HRQoL, fatigue, pacing, risk of overactivity, PA, and self-regulation of PA in adults with chronic conditions and (2) examine if these associations differ across HRQoL domains: physical, social, emotional, and functional well-being.

**Methods:**

Sixty-six adults with chronic conditions were recruited from UK fatigue clinics and the community. HRQoL, pacing, risk of overactivity, PA, and self-regulation of PA were assessed with standardised questionnaires and Actigraph monitor. Associations were analysed with linear mixed models, correcting for confounders.

**Results:**

HRQoL was significantly associated with fatigue (B=-7.82), pacing (B=-0.23), and self-regulation of PA (B = 0.11). Interaction effects revealed fatigue’s impact on HRQoL varied significantly in physical (β=-13.49), social (β=-6.81), and emotional (β=-4.10) domains. Pacing showed significant differences in physical (β=-0.49), social (β=-7.12), and emotional (β=-7.45) domains. Perceived overactivity differed in social domain (β=-6.27), while device-based PA differed in physical (β = 0.35) and social (β = 5.73).

**Conclusion:**

The negative association between fatigue and HRQoL underscores the importance of effective fatigue management. Higher pacing engagement and lower HRQoL may indicate higher fatigue. Positive associations between self-regulation and PA with HRQoL emphasise benefits of appropriate PA behaviours. The stronger impact of decreased fatigue, increased pacing, and PA on physical well-being suggests a multidimensional fatigue management approach.

**Supplementary Information:**

The online version contains supplementary material available at 10.1186/s13102-025-01057-x.

## Background

A wide range of chronic conditions are a significant public health concern [1] and are characterised by symptoms that greatly impact physical function, well-being, and health-related quality of life (HRQoL) [2]. Among these symptoms, fatigue stands out as one of the most prevalent and complex, affecting up to 55% of individuals with chronic conditions [3–5]. In this study, we use a transdiagnostic approach, focusing on fatigue across various chronic conditions, given its common link with numerous chronic conditions [5]. This approach is also helpful when the underlying causes of a condition remain undetermined [6]. Additionally, the prevalence of multimorbidity, defined as the presence of two or more chronic conditions, has risen, with a recent meta-analysis reporting a global prevalence of 37.2% [7]. Multimorbidity complicates the management of a single chronic condition, making transdiagnostic approaches increasingly relevant for effectively addressing common symptoms such as fatigue. Utilizing a transdiagnostic approach in fatigue management provides a more symptom-oriented viewpoint, advancing our understanding of effective management methods [6, 8]. This approach also underscores the significance of symptom-dependency and disease-independency. Notably, existing literature traditionally focused on exploring different chronic conditions separately, while transdiagnostic approaches are now advocated in fatigue and mental health research [9–11]. There are also increasing examples of programs promoting physical activity (PA) transdiagnostically through rehabilitation counselling [12–14]. Therefore, adopting a transdiagnostic approach allows for a more holistic understanding of fatigue management across various chronic conditions, offering potential improvements in HRQoL [8, 15].

Fatigue can reduce PA engagement, with both underactivity and overactivity being linked to disability [16]. When adults with chronic conditions experience fatigue, vigorous exercise can cause negative affective feelings, potentially leading to disengagement from activities for excessive periods [17–20]. Therefore, self-regulation of affective states through activity pacing might be an important determinant of sustained PA behaviour. Activity pacing involves breaking down daily tasks into more manageable segments including periods of rest, allowing individuals to cope with fatigue while maintaining a consistent level of activity, enhancing HRQoL [21–23]. In a recent qualitative study, adults with chronic conditions who experience fatigue perceived engagement in pacing as a forced practice, making them feel restricted [15]. Despite this, they acknowledged that pacing is beneficial for managing fatigue and doing more activities during the day [15]. Moreover, sustained PA offers numerous health benefits for adults with chronic conditions including enhanced HRQoL, functional capacity, muscular strength, and fatigue alleviation [6, 24]. Despite these benefits, PA levels among adults with chronic conditions are lower than in the general population, with approximately 47% of individuals with disabilities being inactive [25]. Therefore, developing strategies to address fatigue symptoms while promoting a sustained physically active lifestyle for adults with chronic conditions is imperative.

Recently, we underscored the multidimensional nature of activity pacing through a conceptual model involving factors including fatigue, PA, social environment, anxiety/depression, rest, and self-regulation, with an overarching goal of enhancing HRQoL [8, 20]. To date, while previous intervention studies have demonstrated the benefits of tailored activity pacing guidance on fatigue management for individuals with chronic conditions [19, 20, 26], they have not yet included all facets of the multidimensional concept [8]. Furthermore, HRQoL is a multidimensional dynamic concept and reflects how individuals perceive and respond to their health status and life aspects encompassing physical, social, emotional, and functional well-being [27, 28]. While factors such as fatigue, PA, and engagement in pacing have been analysed before [29, 30], showing that sustained PA is linked to reduced fatigue and risk of overactivity [29], no study has examined the different domains of HRQoL including physical, social, emotional, and functional well-being in fatigue management and pacing context among chronic conditions and specifically how fatigue, PA, and pacing are associated with specific domains of HRQoL. HRQoL offers a thorough assessment of all health-related aspects of quality of life [31]; therefore, it is important to examine its domains in relation to fatigue management and activity pacing.

Given the negative impact of fatigue in chronic conditions and the potential benefits of activity pacing, PA engagement, and self-regulation as well as fatigue [32], activity pacing [8, 33], and HRQoL [34] are multidimensional concepts, there is a need to delve deeper into our understanding of this multidimensional approach to enhance HRQoL in chronic conditions. This knowledge can inform the development of more effective future interventions in fatigue management [8]. This study takes a distinct cross-sectional approach to build upon prior research and literature acknowledging different factors in pacing and fatigue management for HRQoL enhancement [8, 19, 21, 26]. This comprehensive approach fills a gap in the literature and offers valuable insights into the interrelationships between HRQoL, fatigue, activity pacing, self-regulation, and PA.

Gaining insights into how indices of fatigue and pacing (fatigue, engagement in pacing, risk of overactivity, PA [self-reported & device-based], self-regulation of PA) are associated with HRQoL across different chronic conditions, is significant for researchers and healthcare professionals, adding to previous literature [23, 26, 29, 30, 35–37]. Therefore, this study aimed (1) to examine the associations between HRQoL and indices of fatigue, and activity pacing in adults with chronic conditions, and (2) to examine if these associations differ across the domains of physical, social, emotional, and functional well-being. Based on our expectation that HRQoL is the overarching goal of activity pacing [8, 15], we hypothesised that enhanced HRQoL will be related to decreased fatigue, decreased engagement in pacing, higher PA (self-reported and device-based), higher self-regulation of PA, and decreased risk of overactivity.

## Methods

### Design and ethical approval

This cross-sectional study included 66 participants diagnosed with chronic conditions. The study procedures were approved by the HRA and Health and Care Research Wales REC 4 (IRAS ID: 313465) and by the Northumbria University Ethics Committee (Reference: 3396). The study has been registered at ClinicalTrials.gov (NCT06001970).

### Study population

Individuals with chronic conditions who experience fatigue were recruited through NHS fatigue clinics (currently attending or on a waiting list) and from the community, both in Newcastle Upon Tyne in 2023. Specifically, the potential participants from the fatigue clinics were invited by the clinicians, through a letter and information sheet. Additionally, the community recruitment process involved sending an email with information about the research study to both students and staff members at the university community. In both cases, the participants were asked if they had received advice on fatigue management/activity pacing or not, as from the literature we have seen that receiving fatigue management advice makes a difference in fatigue and PA [19, 35, 37]; thus, this variable was used as a confounder in the analysis named ‘pacing advice’. Potential participants were informed about the study rationale, procedures, risks, and benefits, had any questions answered, and were checked for the inclusion criteria. Participants were included if they: (1) were adults ≥ 18 years of age, (2) were diagnosed with any chronic condition, lasting three months or longer [38], (3) experience fatigue, and (4) were ambulatory. Participants were excluded if they were not able to complete the study questionnaires even with help. Eligible participants who volunteered signed a written informed consent form.

The sample size was estimated a priori using G*Power 3.1.9.7 for linear multiple regression [39]. For a power of 0.80, alpha of 0.05 and an effect size of 0.27, the sample size was estimated to 61 participants. The effect size was derived from a similar study using the same outcome measures [30]. The recruitment target was set to 70 participants to account for attrition of about 15%.

### Procedure

Enrolled participants were assessed through filling out a set of questionnaires digitally. First, participants were asked to answer demographic (e.g., gender, age, employment status) and anthropometric (body mass and height) questions. Then, participants filled out questionnaires on fatigue, activity pacing, HRQoL, self-regulation of PA, and self-reported PA (questionnaires provided in supplementary file 1). Additionally, for this study but not compulsory, participants were invited to wear an Actigraph WGT3X-BT (ActiGraph, Pensacola, USA) for 7 consecutive days.

### Outcome measures

#### Questionnaires

##### Health-related quality of life

Overall HRQoL was assessed by the 27-item Functional Assessment of Cancer Therapy—General Instrument (FACT-G) [40]. The score ranges from 0 to 108 and higher scores indicate better HRQoL. The scale has been validated in several clinical populations [41–44]. FACT-G includes four domains of HRQoL including physical well-being (PWB, seven items), social well-being (SWB, seven items), emotional well-being (EWB, six items), and functional well-being (FWB, seven items). The score ranges from 0 to 28 for PWB, SWB, and FWB and 0–24 for EWB. Higher scores indicate better well-being for each domain. We also used the sub-scales of the FACT-G as effect modifiers in this study.

##### Fatigue

Fatigue severity was assessed by using the 9-item Fatigue Severity Scale (FSS) questionnaire [45], which ranges from 1 to 7 (1 = completely disagree; 7 = completely agree) and is considered a valid and reliable measurement to determine the impact of fatigue in several patient populations [46, 47]. A higher score indicates greater fatigue severity, while a mean score of 4 or greater indicates severe fatigue.

##### Activity pacing and risk of overactivity questionnaire

Activity pacing was assessed by the Activity Pacing and Perceived Risk of Overactivity Questionnaire, which is a seven-item questionnaire developed by the ReSpAct team [48]. In the questionnaire, two different attitudes of activity pacing are assessed: engagement in activity pacing (five items; score ranging 5–25; higher score indicating high engagement in pacing) and perceived risk of overactivity (two items; score ranging 2–10; higher score indicates high risk of perceived overactivity) on a scale ranging from 1 to 5 (1 = never; 5 = very often). The participants scored seven items and the sum scores were calculated for the two different attitudes. Reliability and construct validity have been investigated by the ReSpAct research team and a paper is in preparation [48].

##### Self-regulation of physical activity

Physical Activity Self-Regulation scale (PASR-12) was used to assess self-regulation of PA [49]. PASR-12 consists of 6 domains (2 items each): self-monitoring, goal setting, eliciting social support, reinforcements, time management, and relapse prevention. PASR-12 has demonstrated validity and reliability in adults [50]. The score ranges from 1 to 5 (1 = never; 5 = very often) and total scores were calculated by summing up the scores of the items. Higher scores indicate greater use of self-regulatory strategies.

##### Self-reported physical activity

Self-reported PA was assessed by the International Physical Activity Questionnaire-short form, which includes 7 items [51]. This questionnaire records the intensity and time spent on each PA within the last 7 days. These values are entered into a scoring protocol, to produce either a category (e.g., low, moderate, vigorous) or a continuous variable (e.g., MET minutes per week). Based on the protocol, metabolic equivalent scores were calculated using the following values: low = 3.3 METs, moderate PA = 4.0 METs, and vigorous PA = 8.0 METs. It has been validated in different populations [52, 53]. The continuous variable for self-reported PA METS per week was used for the statistical analysis of this study.

#### Device-based physical activity

Device-based PA was assessed by the Actigraph WGT3X-BT (Pensacola, Florida, USA), which is a reliable, valid, and widely accepted method [54]. The Actigraph WGT3X-BT measures PA in 3 axes and intensity (sedentary, light, moderate, or vigorous). A minimum of 5 consecutive days of monitoring is required for reliably estimating PA from Actigraph data in adults [55]. For each willing participant, one wrist accelerometer was sent by mail to their preferred address between June and October 2023. The participants were advised to wear it on their non-dominant wrist for 7 consecutive days on a normal week for them, 24 h a day, starting at any time of their choosing. This approach ensured that a full 7 days of measurement would be recorded, including one weekend (Saturday and Sunday). All participants were instructed to wear the accelerometer including during sleep, except for when bathing, showering, or swimming, ensuring consistency and accuracy across participants. The device was fully charged when delivered to participants, so they did not need to charge it during the 7-day period. Following 7 days of wear, participants were asked to return their device by mail using an envelope with postage pre-paid.

The device was initialized at a frequency of 30 Hz and accelerometer data were downloaded using ActiLife software 6.6.3 and were analyzed in 60-sec epochs. For the analysis in ActiLife software, data were considered valid only if the participant used the accelerometer for a minimum of 10 h of daily recordings and at least 4 days including a weekend day [56]. Periods with consecutive values of zero (with a 2-min spike tolerance) for 60 min or longer were interpreted as ‘‘accelerometer not worn’’ and were excluded from the analysis [57]. Night-time sleep was excluded by removing the hours between 00:00–06:00 on each day and potential daytime sleep was considered as sedentary time. The time spent in each intensity of PA and sedentary time were estimated based on the cut points proposed by Freedson [58], considering sedentary time as 0 to 99 counts per minute (CPM), light PA as 100 to 1951 CPM, and moderate-to-vigorous PA (MVPA) as ³1952 CPM using the vertical axis and analyzed in minutes/week, adjusting for the number of days and daily hours that the device was worn. For this study, MVPA percentage in those 7 days was considered as an outcome variable for the device-based PA and used for the statistical analysis, which was automatically calculated by ActiLife software based on the cut point algorithm and criteria, we explained above. Although waist-worn accelerometers have been more commonly used to assess PA, wrist-worn accelerometers were specifically chosen to maximize wear compliance [59].

#### Background measures

Demographics including age, gender, education level, body mass, and height were collected in the demographics and anthropometrics questions through an online questionnaire. Overweight and obesity were defined as a body mass index (BMI) of 25–29 and ³30 kg×m^−2^, respectively [60].

### Covariates determination

Referring to previous literature, relevant covariates were considered as potential confounding factors in our analysis [61–63], including socio-demographic characteristics (age, biological sex), lifestyle factors (BMI), and chronic disease conditions (duration of condition, pacing advice [if they have received fatigue management advice] and years of fatigue management advice if received). Ages were recorded and analyzed as individual, continuous values without grouping into categories. Biological sex was dichotomized into male and female. BMI was calculated as body weight in kilograms divided by meters squared and categorized as < 25, 25–29, and ≥ 30. Duration of condition was categorised as 3–6 months, 6–12 months, 1–2 years, and over 2 years. The confounder ‘pacing advice’ was categorised into received fatigue management advice and not received fatigue management advice. For the participants that had received fatigue management advice, the variable ‘years of fatigue management advice’ was categorised into 1–6 months, 6–12 months, 1–5 years, and 5–10 years, while ‘0’ was entered for participants that had not received advice.

### Statistical analyses

Statistical Package for the Social Science version 28 (IBM SPSS Statistics) [64] was used for statistical analyses. The normality assumption was tested using the Shapiro–Wilk test. Normally distributed continuous variables were reported with mean (M) and standard deviation (SD). Non-normally distributed continuous variables were reported using median (Mdn), and interquartile range (IQR). Frequency (n) and percentage (%) were used for the categorical variables to summarize the characteristics of participants. The significance level was set at a *p*-value of < 0.05 for all the statistical analyses in this study.

Since only 29 participants consented to wear the Actigraph activity monitor, we have a large number of missing data values in the variable of device-based PA. To explore the large number of missings, we compared those who completed the device-based PA vs. those who did not to see if there are any significant differences that could influence the interpretation of the findings of this variable. Normally distributed continuous variables were reported with mean (M) and standard deviation (SD) and independent T-test was used to compare the two groups. Non-normally distributed continuous variables were reported using median (Mdn) and interquartile range (IQR) and Mann Whitney U test was used to compare the two groups. Frequency (n) and percentage (%) were reported for the categorical variables and chi-squared test was used to compare the two groups. The significance level was set at a *p* value of < 0.05.

Univariable Mixed Models: First, we performed six univariable mixed model analyses with overall HRQoL (based on the four domains of HRQoL being the repeated measures) as the dependent variable and one of the six indices of fatigue and pacing factors as the independent variable: fatigue, engagement in activity pacing, perceived risk of overactivity, self-regulation of PA, self-reported PA, and device-based PA. Random intercepts at participant level were added. The normality of the residuals was checked by performing linear regression analyses. The residuals of the linear regression model with fatigue and self-reported PA associated with HRQoL were non-normally distributed; therefore, we log-transformed fatigue and self-reported PA and used the log-transformed fatigue and self-reported PA variables in the univariable and multivariable analyses. We also checked for independence of observations, linearity between the independent and dependent variables, and homoscedasticity, and all these assumptions were met.

Multivariable Mixed Models: Second, the six multivariable mixed models were corrected for pacing advice (whether participants had received pacing advice), biological sex, age, BMI, duration of condition, and years of fatigue management advice.

Interaction effects: Third, to examine if the associations between the six independent variables and HRQoL were different across the levels of HRQoL domains (PWB, SWB, FWB, and EWB), we added interaction effects to the mixed models with the PWB being the reference domain. Six different models were performed for each of the independent variables. The estimates of the significant associations between the independent variable and one of the domains of HRQoL were calculated based on the estimates of the independent variable, the estimates of the domains of HRQoL, and the estimates of the interaction-effect variables (see Supplementary Table 1 for detailed calculations).

The linear mixed models have benefits over traditional analysis of variance (ANOVA) as it allows the inclusion of both fixed and random effects and can help minimise type-1 error [65]. Additionally, linear mixed models can help increase statistical power compared to traditional ANOVA and can deal with missing data [66].

## Results

The descriptives of the participants are presented in Table 1. An estimated of 80–120 letters with the information sheet were sent to potential participants in the fatigue clinics, while an email with the study information sheet was sent to a university to all students and staff members at the time. A total of 66 participants (86.4% women), with a median age of 54 (IQR 29) were included in the study and 84.8% of them have experienced fatigue for over 2 years. Participants had a median BMI of 27.4 (IQR 5.8), indicating they were in the overweight range, tending towards obesity. Of these, 25 participants (37.87%) had received fatigue management advice, while most of them (44.0%) had received such advice for 1–5 years. Out of the 66 participants who completed the questionnaires, 29 consented to wear the Actigraph for 7 consecutive days. The median number of valid days of accelerometer data for these participants was 8.00 days. All the participants reported significant fatigue on the FSS (FSS mean score > 4). See supplementary Table 2 for specific diseases of participants.

**Table 1:** Descriptive of participants (*N* = 66)

	All Participants
Number	66
Age (years)	54(29)
Body Mass (kg)	70(16)
Height (cm)	166.52±8.63
BMI (kg×m^−2^)	27.4(5.8)
Valid Actigraph Days	8(0)
Biological Sex	
Female	60 (86.4%)
Male	6 (9.1%)
Employment status	
Full-time	12 (18.2%)
Part-time	11 (16.7%)
Not Employed	11 (16.7%)
Retired	11 (16.7%)
Student	19 (28.8%)
Prefer not to say	2 (3.0%)
Education	
Secondary School	4 (6.1%)
Sixth from College	13 (19.7%)
Vocation Qualification	5 (7.6%)
Undergraduate University	21 (31.8%)
Postgraduate	23 (34.8%)
Marital Status	
Married	25 (37.9%)
Cohabiting	11 (16.7%)
Single	23 (34.8%)
Separated	2 (3.0%)
Divorced	4 (6.1%)
Prefer not to say	1 (1.5%)
Duration of Condition	
3–6 months	2 (3.0%)
6–12 months	2 (3.0%)
1–2 years	6 (9.1%)
Over 2 years	56 (84.8%)
Length of Time Pacing Advice Received (For the group that only received advice n=25)	
1–6 months	9 (36.0%)
6–12 months	3 (12.0%)
1–5 years	11 (44.0%)
5–10 years	2 (8.0%)
Fatigue	6.11(2.11)
Engagement in Activity Pacing	17.67±5.83
Perceived Risk of Overactivity	8(2)
Self-regulation of PA	31.25±10.63
Self-reported PA	3048.51(3009.62)
Device-based PA	12.87±6.45
Overall HRQoL	55.03±17.95
Physical well-being	13.95±6.91
Social Well-being	15.18±5.89
Emotional Well-being	12.67±5.03
Functional Well-being	12.71±6.23

Values presented are M±SD for normally distributed variables, Mdn (IQR) for non-normally distributed variables or N (%) for categorial variables; *BMI *body mass index, *kg *kilograms, *cm *centimetres, *m*^2^ metres squared, *HRQoL *health-related quality of life, *PA *physical activity

Out of the 66 participants in the study, 29 consented to wear the Actigraph, while 37 did not. Significant differences were found between these two groups in age (*p* = < 0.001), employment status (p = < 0.01), marital status (*p* = .041), receipt of pacing advice (*p* = < 0.001), and length of pacing advice (*p* = .005). No significant differences (*p* > .05) were found for body mass, height, BMI, biological sex, education, duration of condition, fatigue, engagement in activity pacing, perceived risk of overactivity self-regulation of PA, self-reported PA, overall HRQoL, physical, social emotional, and function well-being. Demographic characteristics and the comparison of these two groups can be found in supplementary Table 3.

The univariable mixed models (Table 2) revealed significant associations between overall HRQoL and fatigue (β=−9.15; *p* = < 0.001; CI [−12.51, −5.79]), engagement in pacing (β=−0.28; *p* = .002; CI [−0.45, −0.11]), and self-regulation of PA (β = 0.11; *p* = .023; CI [0.02, 0.21]). There were no significant associations found between overall HRQoL and perceived risk of overactivity (β=−0.05; *p* = .875; CI [−0.65, 0.56]), self-reported PA (β = 1.94; *p* = .093; CI [−0.33, 4.23]), and device-based PA (β = 0.15; *p* = .170; CI [−0.07, 0.37]).

The multivariable mixed models (Table 2) revealed significant associations, while corrected for confounders, between overall HRQoL and fatigue (β=−7.82; *p* = < 0.001; CI [−11.33, −4.31]), engagement in pacing (β=−0.23; *p* = .006; CI [−0.39, −0.07]), and self-regulation of PA (β = 1.32; *p* = .013; CI [0.02, 0.20]). There were no significant associations found between overall HRQoL and perceived risk of overactivity (β=−0.12; *p* = .654; CI [−0.66, 0.42]), self-reported PA (β = 1.06; *p* = .342; CI [−1.15, 3.26]), and device-based PA (β = 0.16; *p* = .137, CI [−0.05, 0.37]), while corrected for confounders.

Significant interaction effects were found in the mixed models with fatigue, engagement in pacing, perceived risk of overactivity, and device-based PA (see supplementary Table 1 for specific calculations). The association between overall HRQoL and fatigue is significantly different between the physical (β=−13.49; *p* = < 0.001; CI [−18.5, −8.47]), social (β=−6.81; *p* = < 0.001; CI [5.72, 17.59]), and emotional (β=−4.10; *p* = .023; CI [0.94, 12.82]) well-being domains of HRQoL. The association between overall HRQoL and engagement in pacing is significantly different between the physical (β=−0.49; *p* = < 0.001; CI [−0.73, −0.27]), social (β=−7.12; *p* = < 0.001; CI [0.23, 0.77]), and emotional (β=−7.45; *p* = .008; CI [0.09, 0.64]) well-being domains of HRQoL. The association for the functional domain was non-significant (*p* > .05). The association between overall HRQoL and perceived risk of overactivity is significantly different for the social (β=−6.27; *p* = .013; CI [0.24, 2.07]) well-being domain of HRQoL. The associations between the functional, physical and emotional domains were non-significant (*p* > .05). The association between HRQoL and device-based PA is significantly different between the physical (β = 0.35; *p* = .021; CI [0.56, 0.65]) and social (β = 5.73; *p* = .002; CI [−0.91, −0.21]) well-being domains of HRQoL. The associations between the functional and emotional domains were non-significant (*p* > .05).

**Table 2:** Associations between indices of fatigue and pacing using univariable and multivariable linear mixed models with overall HRQoL as dependent variable

Variables	N	β	95% CI		p	β	95% CI		p
		**Univariable Mixed Models**				Multivariable Mixed Models**			
Fatigue	66	−9.15	−12.51	−5.79	<0.001*	−7.82	−11.33	−4.31	<0.001*
Engagement in Pacing	66	−0.28	−0.45	−0.11	0.002*	−0.23	−0.39	−0.07	0.006*
Perceived Risk of Overactivity	66	−0.05	−0.65	0.56	0.875	−0.12	−0.66	0.42	0.654
Self-regulation of PA	65	0.11	0.02	0.21	0.023*	0.11	0.02	0.20	0.013*
Self-reported PA	60	1.94	−0.33	4.23	0.093	1.06	−1.15	3.26	0.342
Device-based PA	29	0.15	−0.07	0.37	0.170	0.16	−0.05	0.37	0.137

*p<0.05; **Models were corrected for pacing advice, biological sex, age, BMI, duration of condition, and years of fatigue management advice; *β *standardized regression coefficient, *N *number

## Discussion

The present study examined the associations between overall HRQoL and various independent variables, including fatigue, PA (self-reported and device-based), self-regulation of PA, engagement in pacing, and perceived risk of overactivity in adults with chronic conditions. Demographic variables such as gender, age, BMI, duration of the condition, self-reported receiving of fatigue management advice, and years of receiving advice or not were considered as confounders. In line with our hypothesis, the findings revealed significant associations between overall HRQoL and fatigue, engagement in pacing, and self-regulation of PA, while corrected for confounders. Notably, significant interaction effects (domains of HRQoL: PWB, SWB, EWB) were found in the models with fatigue, engagement in pacing, perceived risk of overactivity, and device-based PA. Lower fatigue, more engagement in pacing, and higher device-based PA have a greater impact on the physical subscale of HRQoL compared to the other subscales. Decreased perceived risk of overactivity and higher device-based PA have a stronger association with social subscale of HRQoL compared to the other subscales.

Higher fatigue is associated with decreased HRQoL, while corrected for confounders, aligning with the findings of Abonie et al. in multiple sclerosis population [30], revealing a negative association between fatigue and HRQoL. Similar associations between fatigue and HRQoL have also been observed in bowel disease, systemic lupus erythematosus, and sjögren’s syndrome [67–69], underscoring the crucial role of effective fatigue management in chronic conditions to improve HRQoL. Furthermore, this finding emphasizes the common presence of fatigue across diagnostic categories and underscores the necessity for optimal fatigue management interventions that can benefit individuals experiencing fatigue [8], especially when the underlying causes remain undetermined [6]. A meta-analysis among chronic conditions with fatigue symptoms found that activity pacing interventions had medium effects on fatigue management and reduction [23]. Moreover, higher fatigue is associated with decreased physical, social, and emotional well-being. Particularly, the negative association between decreased fatigue and the physical, social, and emotional well-being domains is stronger for the physical domain of HRQoL compared to the other domains. A study has shown that fatigue impacts physical and psychological function, walking ability, and depression in multiple sclerosis [70]. Furthermore, another study found that fatigue is associated with decreased participation in social roles and activities in systemic sclerosis [71]. Therefore, the association between the overall HRQoL and fatigue is similar to the association between fatigue and HRQoL’s different domains. These findings emphasize the need for effective interventions to address fatigue as a cross-cutting issue across different domains of HRQoL. Addressing the underlying mechanisms of fatigue, such as inflammatory pathways, central sensitization, and psychological factors, may further clarify its pervasive impact across conditions [72, 73]. Fatigue is common in inflammatory diseases, such as rheumatoid arthritis, where it is associated with disease activity, inflammation, and psychological factors [72]. Biologic agents targeting inflammatory cytokines can reduce fatigue to some extent by addressing inflammation [72]; however, their effect is often small [74, 75]. For instance, a meta-analysis in inflammatory bowel disease demonstrated a small but consistent reduction in fatigue with biological and small molecule agents, highlighting that fatigue persists despite treatment [75]. Similarly, in psoriatic arthritis, a global study showed that substantial fatigue and pain persist even with biological therapies, contributing to reduced HRQoL and work productivity [74]. These findings underscore the need for non-pharmacological interventions to comprehensively address fatigue and improve overall well-being in these conditions. On the other hand, central sensitisation involves various processes that increase the central nervous system’s sensitivity to stimuli, and its role in chronic fatigue is linked to psychological factors [73]. Therefore, understanding and targeting the mechanisms of fatigue is crucial for improving overall well-being in chronic conditions.

Similarly to our initial hypothesis, more engagement in pacing was associated with decreased HRQoL, after correcting for confounders. This finding could be explained by the fact that the need to pace is linked to higher fatigue [15] and ultimately decreased HRQoL. Specifically, more engagement in pacing was associated with decreased physical, social, and emotional well-being domains of HRQoL compared to functional well-being in the interaction effect models, with the physical domain having a stronger association compared to other HRQoL domains. Contradictory to our findings, a systematic review concluded that the use of pacing is associated with less negative emotions in chronic pain [36]. This discrepancy might be explained by several factors. First, it is important to note that while higher engagement in pacing might alleviate negative emotions, it does not necessarily enhance the overall emotional well-being as negative emotions are different from emotional well-being. Second, the experience of fatigue, as examined in our study, may differ from the experience of pain, even though they have much in common [76]. These findings highlight the complexity of pacing behaviors and suggest the need for further research to better understand the relationships between engagement in pacing, and emotional well-being in chronic fatigue but also chronic pain. Insights from cancer-related fatigue interventions suggest that combining pacing with psychological approaches, such as mindfulness or cognitive-behavioural therapy, may provide additional benefits for emotional well-being [77].

Consistent with our hypothesis, higher self-regulatory skills in PA were associated with higher HRQoL. This finding supports the development of interventions that focus on enhancing self-regulatory skills to improve HRQoL as self-regulation is a key mechanism in health behaviour change interventions [78]. Furthermore, the significance of self-regulatory skills in PA is underscored by their potential to promote sustained moderate to vigorous engagement in PA, which is relevant for addressing fatigue complaints [79, 80]. These skills have been recognised as pivotal in health behavioural theories, known for their contribution to exercise regulation and potential to improve health [81]. For example, a study conducted among cancer patients implemented a PA intervention that highlighted the importance of self-regulatory skills within the active control group, leading to consistent and prolonged engagement in PA [82]. Our findings, along with the literature, underline the importance of interventions incorporating self-regulation education, wherein patients, for example, are taught to pre-plan and set goals [83]. Such interventions have the potential to improve not just physical health, but also mental and emotional well-being. Because PA involves complex planning, monitoring, ongoing adjustments, and inhibition of unwanted distractions [84], such interventions can boost health-related benefits like reduced fatigue, improved mental and emotional well-being, and sustained PA participation. Moreover, individuals with chronic conditions, experiencing fatigue, expressed the need to learn when and how to slow down, as they often do not know when to stop [15], leading to overactivity and post-exertional malaise [85]. This underscores the critical need for patient education in pacing and activity management. Similarly, stroke survivors emphasised the need for guidance on activity pacing to effectively manage PA and fatigue [86]. Notably, learning how to regulate their PA and/or exercise is crucial for sustained engagement in PA, given the positive correlation between exercise enjoyment and motivation [87]. More specifically, through pacing, negative experiences related to high-intensity exercise and fatigue may be reduced. Therefore, individuals are more likely to participate in PA/exercise and remain engaged if they find it enjoyable [88], highlighting the potential benefits of pacing advice for promoting PA habit frequency and the intention to stay active [89]. Hence, interventions should encompass multidimensional factors to comprehensively manage symptoms. By incorporating pacing advice and considering factors like enjoyment of PA, interventions may have the potential to not only manage symptoms effectively but also to enhance overall HRQoL.

Furthermore, our study revealed that decreased perceived risk of overactivity is associated with higher social well-being, while there was no association with the overall HRQoL. Previously, a physically active lifestyle was associated with decreased perceived risk of overactivity in individuals after stroke [29]. Perhaps, individuals who perceive themselves to be at risk of overactivity overdo their activities and therefore experience less well-being in social activities. Moreover, linked to the enjoyment of PA, if they have a high perceived risk of overactivity, they might enjoy PA less, which might potentially lead to decreased social well-being. This finding is consistent with qualitative research that suggests individuals who overestimate their physical limits may experience greater fatigue, which could negatively impact social participation and overall well-being [15]. Also, the subsequent recovery time from overactivity might be in the way of their social activities. In some cases, the recovery time may exacerbate feelings of isolation and frustration, further influencing social interactions [90]. This finding emphasizes the necessity of implementing optimal and tailored PA interventions. The importance of personalized pacing strategies in conditions, has been highlighted in the literature [8], showing that individualized interventions can improve both physical and psychological well-being [19]. These interventions may consider individualised strategies including addressing social needs and experiences. Further research is needed to explore how overactivity affects social participation and its long-term impact on well-being, as no studies yet have explored this association.

Moreover, higher device-based PA was associated with higher physical and social well-being with physical domain having a stronger association compared to other HRQoL domains. These findings line up with the literature as sustained PA engagement can help individuals with chronic conditions cope with their symptoms, improving HRQoL and well-being [91]. Specifically, adults with chronic conditions reported that they need to participate in social groups [15], therefore, being physically active in groups could help them meet more people and improve their social well-being by building relationships with others that have common interests. Recent studies have shown that group-based physical activity not only improves physical health but also fosters a sense of community, which is essential for mental health in individuals with chronic diseases [92–94]. Research indicates that individuals with multiple chronic conditions often have a decreased social well-being [95]. Other studies found that social interactions within PA contexts are crucial for mental health benefits [96, 97]. Additionally, research has shown that sustained PA engagement may improve muscle strength and physical functioning [98]. This improvement in physical functioning may also lead to increased self-esteem, enhancing social interactions and overall quality of life [99]. Consequently, chronic disease management programs may incorporate strategies to enhance social well-being within PA engagement. Notably, device-based measures of PA, such as those from accelerometers, tend to be more accurate and precise compared to self-reports, which can be influenced by recall bias and perception [100, 101]. However, device-based PA may not capture all modes of PA, in contrast, self-report measures can inquire about a broader range of activities [102]. Usually in research, both measures are used together to get a more comprehensive view of PA behaviours [102]. Research has shown that both self-reported and device-based PA are linked to higher HRQoL scores [103]. Though, device-based measures tend to have a stronger association with overall HRQoL compared to self-reported PA [103]. Combining both measures may offer a more complete picture of physical activity and its effects on well-being. Another important point is to consider the differences between participants who completed the device-based PA and those who did not. Only 29 participants consented to wear the Actigraph, while 37 did not, resulting in a smaller sample size for device-based PA variable, which may affect the study’s power. Significant differences between these two groups were found in age, employment status, marital status, and receipt of pacing advice. The 29 participants who completed the device-based PA were significantly older and most of them were retired, which may suggest different motivations and more available time to engage in the research compared to younger participants. Research indicates that physical activity, both exercise and leisure-time, tends to increase following retirement [104]. Therefore, such factors may impact their engagement in PA and their HRQoL outcomes, highlighting the importance of considering these variables when interpreting results. Additionally, a larger proportion of the 29 participants received pacing advice compared to the 37 who did not, possibly indicating that those who completed the device-based PA were more informed, motivated or familiar with similar research projects. The differences between these two groups could potentially influence the association between device-based PA and HRQoL, as the results may partly reflect the characteristics of a more specific subgroup rather than the entire study population. Therefore, findings should be interpreted with caution and further research is needed to understand which individuals are more likely to consent to and engage in more demanding research projects.

### Implications and strengths

Due to the cross-sectional nature of this study, conclusions cannot be drawn but our findings may indicate that individuals with chronic conditions turn to pacing as a coping mechanism when experiencing significant fatigue, supported by participants reporting a median FSS score of 6.11 indicating clinically significant fatigue. This suggests a potential area for intervention development, focusing on effective pacing strategies to manage fatigue. Similar conclusions were made in other cross-sectional studies in multiple sclerosis [30, 35]. Moreover, a study that interviewed individuals with chronic conditions further revealed that although pacing helps them achieve more during the day, it is perceived as a mandatory strategy rather than a lifestyle adjustment [15]. This perception highlights the need for interventions that integrate pacing as a positive lifestyle change rather than a necessity, while it is important as it emphasizes that healthcare interventions should aim not just to reduce fatigue but also to help individuals feel empowered in adopting pacing strategies as a natural part of their routine. Building on the implications of this cross-sectional study, the integration of self-regulation strategies, tailored pacing advice, and lifestyle modifications into intervention programs might be essential for effectively managing fatigue and enhancing HRQoL in chronic conditions [8]. The study’s findings advocate for a holistic approach that combines PA with psychological support, such as cognitive-behavioural therapy or mindfulness, to address both the physical and emotional facets of fatigue [8, 15, 19, 20]. This aligns with previous research highlighting the benefits of comprehensive interventions that target multiple dimensions of well-being [8, 15, 20]. Additionally, the use of device-based measures for physical activity can provide more accurate insights into patient behavior and facilitate tailored intervention strategies that address individual needs. Emphasizing patient education on self-regulation and pacing as lifestyle adjustments rather than mere coping mechanisms can empower individuals to take proactive roles in their health management [15]. Consequently, by adopting a multidimensional framework, healthcare providers can develop tailored management plans that not only address the complex nature of chronic fatigue and its impact on daily living but also assist individuals in improving their physical and emotional well-being, ultimately enhancing HRQoL. This suggests that healthcare providers should consider a more holistic approach, integrating not just PA strategies but also addressing the psychological aspects of fatigue management and pacing. Activity pacing approach could be viewed as a lifestyle adjustment [105], recognising that individuals experiencing fatigue often struggle with accepting life changes.

Previous studies have primarily focused on the associations of activity pacing and PA in specific condition groups such as multiple sclerosis and osteoarthritis [30, 106]. Therefore, this study contributes to understanding fatigue symptoms across different conditions, considering the commonness of it. A transdiagnostic approach could offer potential benefits for individuals with significant fatigue and enhance fatigue management approaches for healthcare professionals as well as the development of interventions for fatigue management and improving HRQoL in chronic conditions. For instance, clinicians may consider incorporating self-regulation techniques (such as teaching patients how to monitor their energy levels and set realistic goals) and fatigue management strategies. Research suggests that self-regulation skills can reduce fatigue but also improve emotional well-being by enhancing an individual’s sense of control over their condition [107, 108]. Also, mental health support into their practice could be important, addressing the multidimensional nature of fatigue. Cognitive behavioural therapy, mindfulness, relaxation therapy, and educational counselling have been effective in reducing fatigue in multiple sclerosis [109]. These suggestions would require a multidisciplinary team of healthcare professionals. Rehabilitation interventions, often multidisciplinary and individualized, have shown positive effects on fatigue severity in multiple sclerosis [110]. Individuals with chronic conditions have also reported a need for a multidisciplinary approach to disease management [15]. This reinforces the importance of healthcare teams working together to create tailored intervention plans that address both physical and psychological aspects of fatigue. The findings of this study, along with existing literature [8, 15, 111], may be used by healthcare providers to facilitate more informed and productive conversations with their patients about the multidimensionality of fatigue, activity pacing, and HRQoL. These conversations could help normalize the use of pacing as part of a comprehensive fatigue management strategy, empowering patients to take a proactive role in their own health management. Yet, future research for causal links and interventions is important.

Moreover, the novel inclusion of interaction effects may add depth to the analysis and allow for a more detailed comprehension of how indices of fatigue and pacing may be associated with the different domains of HRQoL. Understanding these interactions is critical, as it could help in designing interventions that target specific HRQoL domains, making interventions more precise and personalized. Furthermore, the influence of lifestyle factors like physical activity, nutrition, and sleep on HRQoL and fatigue management in chronic conditions is pivotal for a comprehensive understanding of patient well-being [8, 112]. Physical activity enhances cardiovascular fitness, muscle strength, and overall endurance, which are essential for handling the physical demands and fatigue associated with chronic conditions [113–115]. Adequate nutrition provides the energy and nutrients required for optimal recovery and overall health maintenance [116, 117]. Specifically, clinical research indicates that consuming a balanced diet containing whole grains high in fiber, vegetables rich in polyphenols, and omega-3 fatty acid-rich foods may contribute to reducing disease-related fatigue symptoms [118]. Quality rest, including both sleep and restful activities, is vital for physical and mental recovery, as well as cognitive function [8, 119, 120]. Specifically, sleep quality and duration are critical for maintaining physical health and cognitive function [121]. Furthermore, restful activities have been reported by individuals with chronic conditions to help them with fatigue management [15]. Recent research underscores the significant roles these factors play in health outcomes and disease management. For example, the relationship between physical activity and immune function can affect recovery and overall health in patients [63, 122], and nutrition has been linked to immune response and treatment efficacy. Therefore, future studies and clinical trials may incorporate these lifestyle factors to provide more comprehensive insights into effective disease/fatigue management and improvement of HRQoL. The integration of these lifestyle factors into fatigue management programs could offer a more holistic approach to improving patient outcomes in chronic conditions.

#### Limitations

Potential limitations should be acknowledged. Firstly, the cross-sectional design limits the ability to establish causal relationships, meaning that while associations are identified, the directionality and causality of these relationships remain uncertain. This limitation suggests a need for longitudinal studies to explore these relationships further. Secondly, drug use information was not collected from participants; however, including it as a confounder in future studies would be valuable. Drug use could have a significant impact on HRQoL, emotional and physical well-being, and fatigue management [123]. Future research should address this gap by collecting comprehensive data on medication usage. Thirdly, the small sample size on the device-based PA variable (*n* = 29) also presents a limitation, possibly affecting the statistical power and generalizability of the findings related to device-based PA. Fourthly, although this study included the independent variables of fatigue, pacing, self-regulation, and PA, more variables play a role in influencing the HRQoL including built environment, nutrition and rest [8]; thus, the findings of this study should be interpreted with caution due to the various factors that can impact HRQoL. These variables should be considered in future studies to provide a more comprehensive understanding of HRQoL. Additionally, this study was conducted only in one region of the United Kingdom, which may limit its generalizability to other regions or countries. Cultural differences in healthcare practices and lifestyle could influence the findings [124], suggesting a need for studies across diverse populations to enhance generalizability. Lastly, despite the inclusion of both females and males in the study, the majority of the sample comprised females. Considering research suggests that adults with chronic conditions experiencing fatigue as a symptom have a female preponderance [125], this may not be surprising; however, future research could explore equal representation of both genders to potentially identify any similarities or differences between the groups.

Future studies should examine activity pacing interventions for fatigue management, including social and emotional support and explore pacing behaviours in chronic conditions in depth. These studies could provide critical insights into developing effective, multidimensional interventions. Moreover, the associations found in this study should be further investigated to understand underlying mechanisms and causal relationships. The findings of this study along with the literature could be used to facilitate discussions between clinicians and their patients regarding the multidimensionality of fatigue management and activity pacing, enhancing patient-provider communication and intervention development. Effective patient-provider communication is essential in healthcare, as it aims to enhance the patient’s health outcomes and foster a trusting relationship between the patient and the doctor [126].

## Conclusion

In this study, we explored the relationships between HRQoL and indices of fatigue and pacing in chronic conditions. We also examined the different domains of HRQoL (physical, social, emotional, and functional well-being) to better understand its multidimensionality. Higher HRQoL were associated with decreased fatigue, pacing engagement, and higher self-regulatory skills, while correcting for confounders. The associations between decreased fatigue, more engagement in pacing, and higher PA with HRQoL are stronger for the physical domain of HRQoL compared to social, functional, and emotional well-being domains. Higher perceived risk of overactivity and device-based PA are associated with higher social well-being. These findings further underscore the importance of fatigue management through a multidimensional approach as fatigue was associated with overall HRQoL but also physical, social, and emotional well-being. Future research should focus on multidimensional interventions for fatigue management, including mental health support and social support, and on understanding better the pacing behaviors in adults with chronic conditions.

## Supplementary Information

Supplementary Material 1

Supplementary Material 2

Supplementary Material 3

Supplementary Material 4

## Data Availability

The datasets used and/or analysed during the current study are available from the corresponding author on reasonable request.

## Abbreviations

HRQoL: Health-Related Quality of Life
PA: Physical activity
FACT-G: Functional Assessment of Cancer Therapy—General Instrument
PWB: Physical Well-being
SWB: Social Well-being
EWB: Emotional Well-being
FWB: Functional Well-being
FSS: Fatigue Severity Scale
PASR-12: Physical Activity Self-Regulation scale
MET: Metabolic Equivalent
cpm: counts per minute
MVPA: Moderate-Vigorous Physical Activity
BMI: Body Mass Index
CI: Confidence Interval
ME/CFS: Myalgic Encephalomyelitis/Chronic Fatigue Syndrome
cm: centimetres
kg: kilograms
