# Supplementary figures and images for: Health-related quality of life associated with fatigue, physical activity and activity pacing in adults with chronic conditions
Source: BMC Sports Sci Med Rehabil. 2025 Jan 28;17:13. doi: 10.1186/s13102-025-01057-x (PMC11773964; doi:10.1186/s13102-025-01057-x)

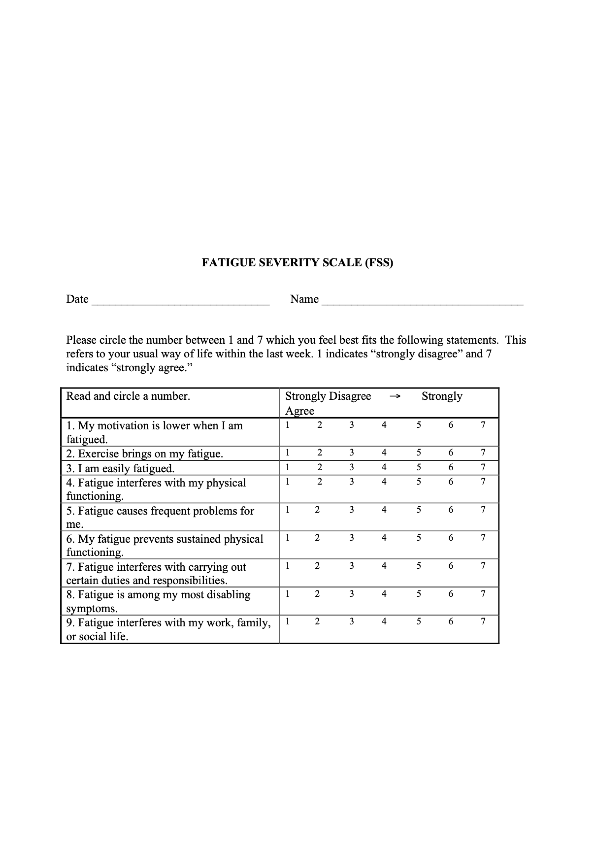


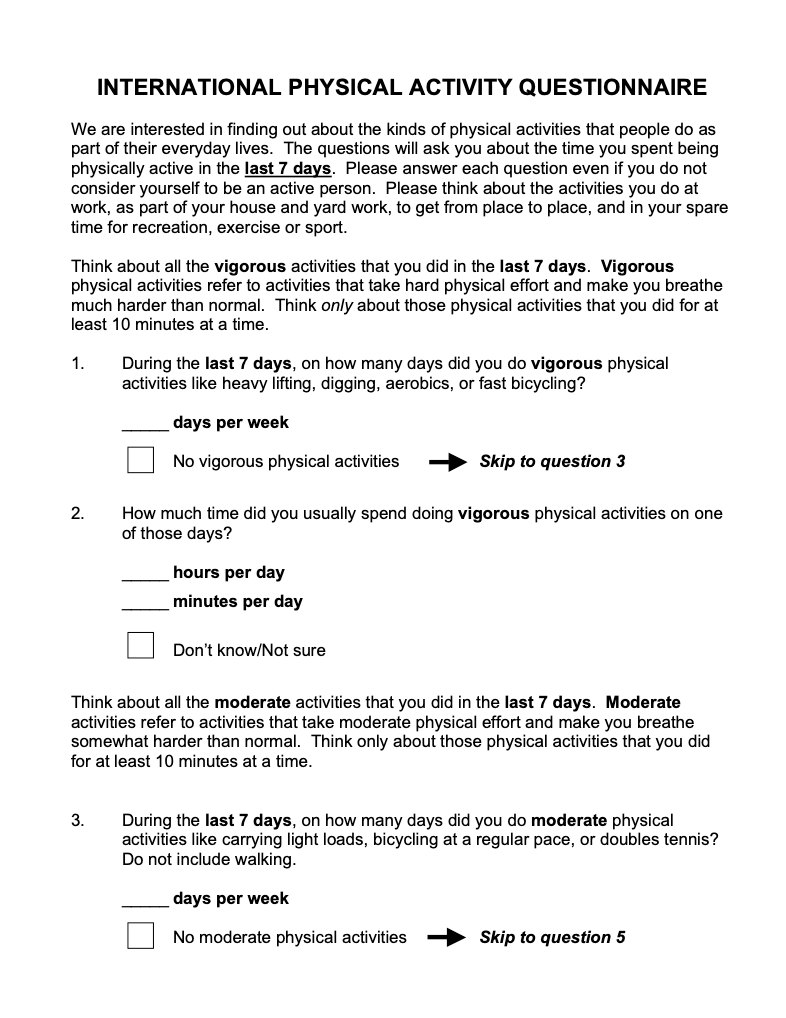


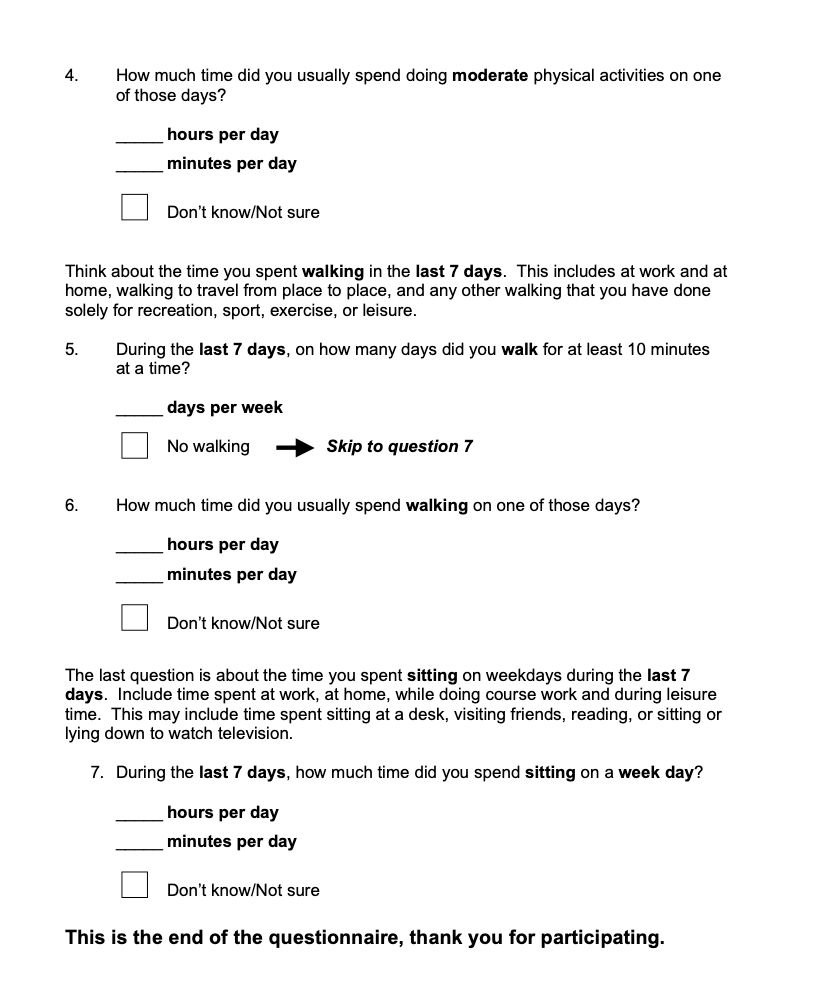


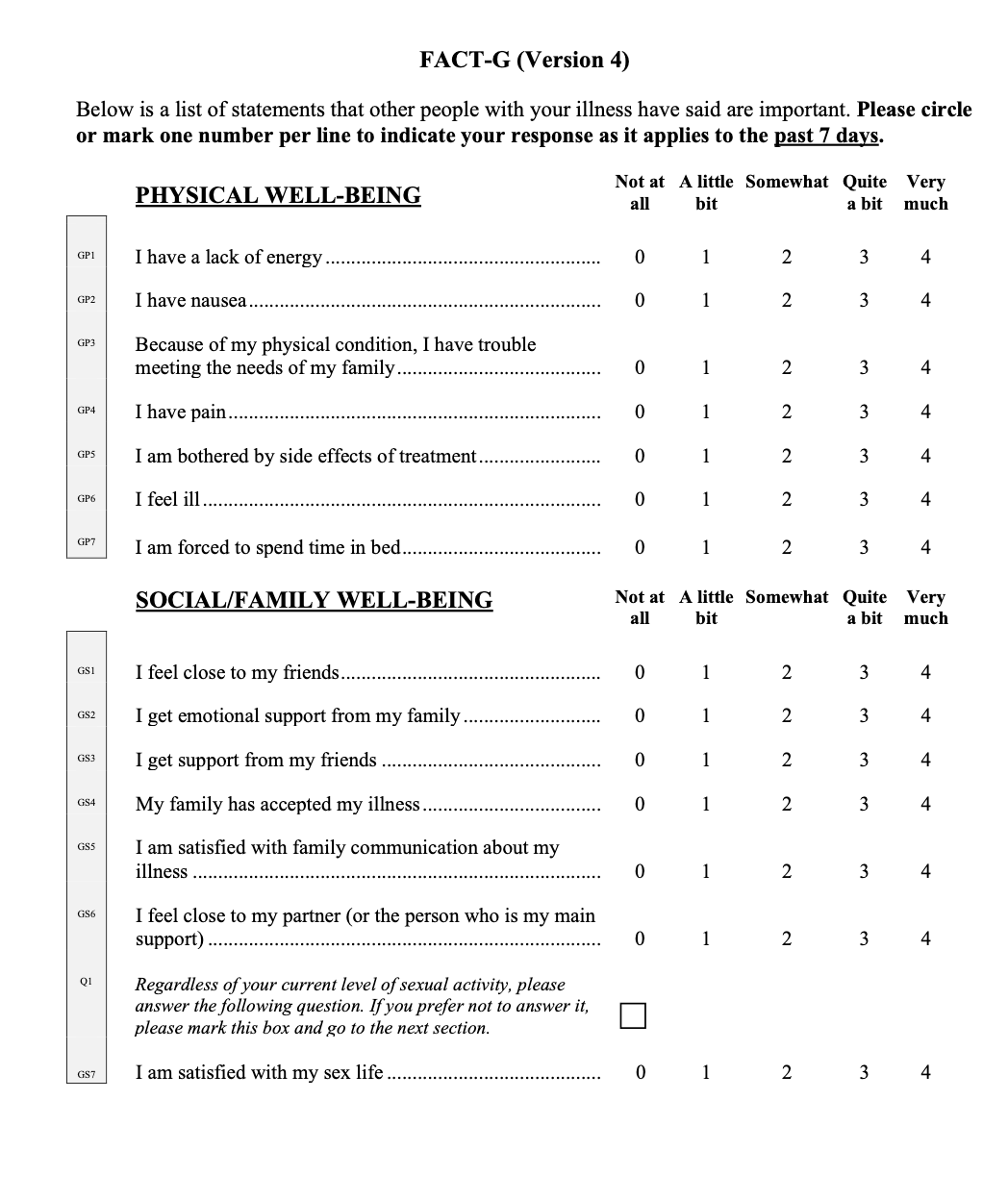


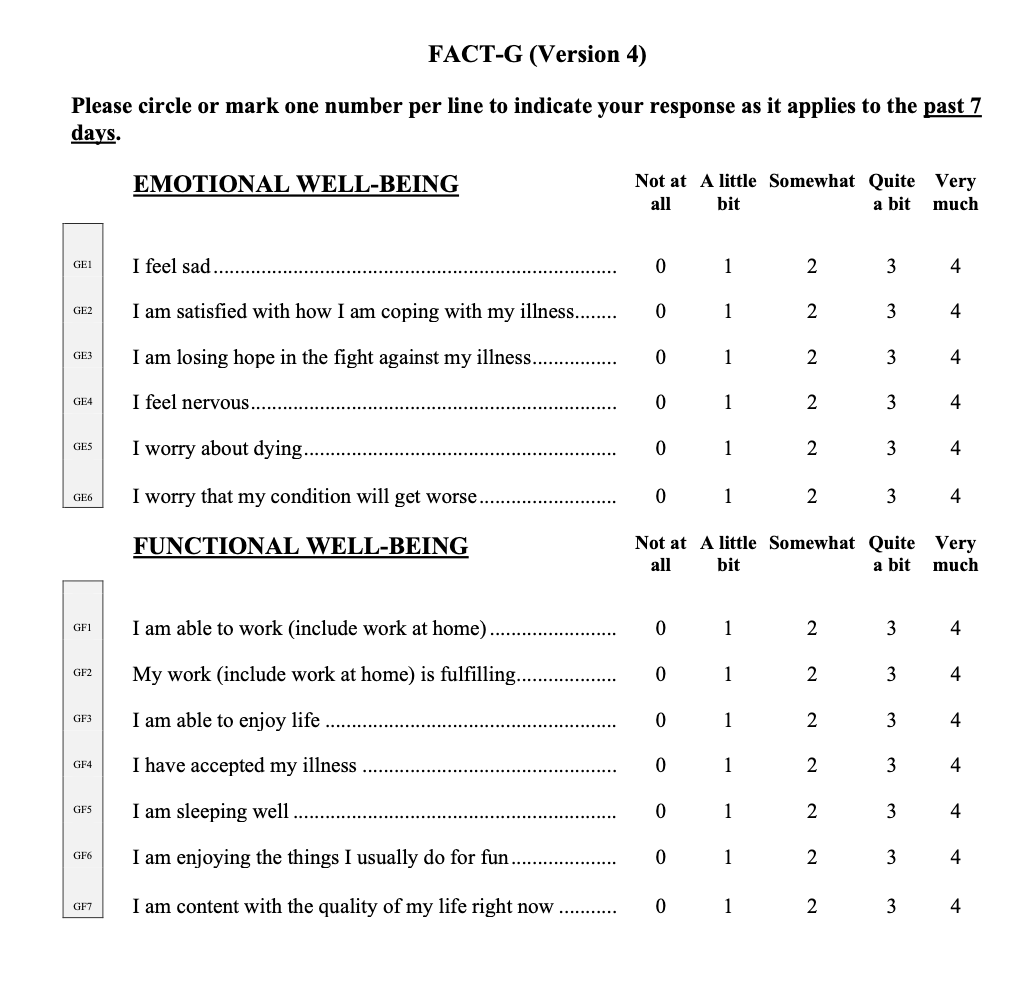

Supplement: Supplementary file 1 — Supplementary Material 1 [file 13102_2025_1057_MOESM1_ESM.docx]
