# Supplementary material for: Health-related quality of life associated with fatigue, physical activity and activity pacing in adults with chronic conditions
Source: BMC Sports Sci Med Rehabil. 2025 Jan 28;17:13. doi: 10.1186/s13102-025-01057-x (PMC11773964; doi:10.1186/s13102-025-01057-x)
Supplement: Supplementary file 2 — Supplementary Material 2 [file 13102_2025_1057_MOESM2_ESM.docx]

Supplementary Table 1. Interaction Effects Mixed Models

| Variables |  | β | 95% CI | | p |
| --- | --- | --- | --- | --- | --- |
|  | | | | | |
| Fatigue |  | -13.49 | -18.51 | -8.47 | <0.001 |
| SWB |  | -18.47 | -28.62 | -8.31 | <0.001 |
| EWB |  | -12.92 | -23.07 | -2.76 | 0.013 |
| FWB |  | -8.24 | -18.40 | 1.91 | 0.111 |
| SWB*fatigue |  | 11.66 | 5.72 | 17.60 | <0.001 |
| EWB*fatigue |  | 6.88 | 0.94 | 12.82 | 0.023 |
| FWB*fatigue |  | 4.14 | -1.79 | 10.08 | 0.170 |
|  |  |  |  |  |  |
| Engagement in Pacing |  | -0.49 | -0.73 | -0.27 | <0.001 |
| SWB |  | -7.62 | -12.69 | -2.54 | 0.003 |
| EWB |  | -7.81 | -12.89 | -2.74 | 0.003 |
| FWB |  | -5.04 | -10.12 | 0.04 | 0.052 |
| SWB* Engagement in Pacing |  | -0.50 | 0.23 | 0.77 | <0.001 |
| EWB* Engagement in Pacing |  | 0.369 | 0.09 | 0.64 | 0.008 |
| FWB* Engagement in Pacing |  | 0.21 | -0.06 | 0.488 | 0.122 |
|  |  |  |  |  |  |
| Perceived risk of overactivity |  | -0.75 | -1.52 | 0.02 | 0.057 |
| SWB |  | -7.43 | -14.43 | -0.42 | 0.038 |
| EWB |  | -5.83 | -12.83 | 1.17 | 0.102 |
| FWB |  | -6.85 | -13.85 | 0.15 | 0.055 |
| SWB* Perceived risk of overactivity |  | 1.16 | 0.24 | 2.07 | 0.013 |
| EWB* Perceived risk of overactivity |  | 0.61 | -0.30 | 1.52 | 0.191 |
| FWB* Perceived risk of overactivity |  | 0.75 | -0.16 | 1.66 | 0.106 |
|  |  |  |  |  |  |
| Self-regulation of PA |  | 0.04 | -0.09 | 0.17 | 0.521 |
| SWB |  | -2.58 | -7.73 | 2.56 | 0.323 |
| EWB |  | -2.84 | -7.98 | 2.31 | 0.278 |
| FWB |  | -5.00 | -10.15 | 0.14 | 0.056 |
| SWB* Self-regulation of PA |  | 0.12 | -0.03 | 0.28 | 0.130 |
| EWB* Self-regulation of PA |  | 0.05 | -0.11 | 0.20 | 0.537 |
| FWB* Self-regulation of PA |  | 0.12 | -0.03 | 0.27 | 0.127 |
|  |  |  |  |  |  |
| Self-reported PA |  | 2.65 | -0.39 | 5.70 | 0.087 |
| SWB |  | 6.05 | -5.60 | 17.71 | 0.307 |
| EWB |  | 6.98 | -4.67 | 18.64 | 0.239 |
| FWB |  | 12.42 | 0.77 | 24.08 | 0.037 |
| SWB* Self-reported PA |  | -1.17 | -4.63 | -2.29 | 0.506 |
| EWB* Self-reported PA |  | -0.00 | -0.00 | 0.00 | 0.817 |
| FWB* Self-reported PA |  | -3.15 | -6.61 | 0.32 | 0.075 |
|  |  |  |  |  |  |
| Device-based PA |  | 0.35 | 0.05 | 0.65 | 0.021 |
| SWB |  | 6.29 | 1.28 | 11.31 | 0.014 |
| EWB |  | -2.07 | -7.09 | 2.95 | 0.414 |
| FWB |  | -1.23 | -6.25 | 3.79 | 0.627 |
| SWB* MVPA%(device-based) |  | -0.56 | -0.91 | -0.21 | 0.002 |
| EWB* Device-based PA |  | -0.00 | -0.35 | 0.35 | 0.988 |
| FWB* Device-based PA |  | -0.21 | -0.56 | 0.14 | 0.236 |

Calculation (β of Domain + (β of Domain x the variable)); Reference domain PWB; β, standardized regression coefficient; CI, confidence interval; SWB, social-wellbeing; EWB, emotional wellbeing; FWB, functional wellbeing; PWB, physical wellbeing; PA, physical activity
