# Supplementary material for: Health-related quality of life associated with fatigue, physical activity and activity pacing in adults with chronic conditions
Source: BMC Sports Sci Med Rehabil. 2025 Jan 28;17:13. doi: 10.1186/s13102-025-01057-x (PMC11773964; doi:10.1186/s13102-025-01057-x)
Supplement: Supplementary file 3 — Supplementary Material 3 [file 13102_2025_1057_MOESM3_ESM.docx]

Supplementary Table 2. Specific Diseases of Participants

| N (%) | | All participants (N=66) |
| --- | --- | --- |
| Comorbidity* | 37 (56.1%) | |
| Addison's disease | 1 (1.5%) | |
| Antiphospholipid syndrome | 1 (1.5%) | |
| Aplastic anemia | 1 (1.5%) | |
| Atopic asthma | 1 (1.5%) | |
| Back pain | 1 (1.5%) | |
| Bowel perforation | 1 (1.5%) | |
| Bronchiectasis | 1 (1.5%) | |
| ME/CFS | 3 (4.5%) | |
| Chronic gallbladder disease | 1 (1.5%) | |
| Coeliac disease | 1 (1.5%) | |
| Ehlers-danlos syndrome | 2 (3.0%) | |
| Fibromyalgia | 1 (1.5%) | |
| Frontal lobe seizures | 1 (1.5%) | |
| hypothyroidism | 1 (1.5%) | |
| Long Covid | 1 (1.5%) | |
| Multiple sclerosis | 4 (6.1%) | |
| Polycystic ovary syndrome | 1 (1.5%) | |
| Rheumatoid arthritis | 3 (4.5%) | |
| Sjogren's syndrome | 3 (4.5%) | |

Values presented are N (%); N, number; ME/CFS, myalgic encephalomyelitis/chronic fatigue syndrome; *Comorbidities (two or more conditions in combination) include combinations of the following: fibromyalgia, postural orthostatic tachycardia syndrome, chronic vertiginous migraine, myalgic encephalomyelitis/chronic fatigue syndrome, type 2 diabetes, hypertension, depression, fatty liver disease, osteoarthritis, autonomic failure, asthma, restless legs syndrome, functional neurological disorder, irritable bowel syndrome, gastroesophageal reflux disease, hypermobile spectrum disorder, postural tachycardia syndrome, Ehlers-danlos syndrome, chronic costochondritis, Raynaud’s syndrome, temporomandibular disorder, chronic gastric volvulus, Sjögren's syndrome, blood clotting disorder, hypermobility spectrum disorder
