# Supplementary material for: Health-related quality of life associated with fatigue, physical activity and activity pacing in adults with chronic conditions
Source: BMC Sports Sci Med Rehabil. 2025 Jan 28;17:13. doi: 10.1186/s13102-025-01057-x (PMC11773964; doi:10.1186/s13102-025-01057-x)
Supplement: Supplementary file 4 — Supplementary Material 4 [file 13102_2025_1057_MOESM4_ESM.docx]

Supplementary Table 3. Comparison of participants who participated in the device-based physical activity measurement vs those who did not

|  | Participants participated in the device-based physical activity measurement | Participants who did not | p |
| --- | --- | --- | --- |
| Number | 29 | 37 |  |
| Age (years) | 54(30.50) | 31.50(22.25) | <0.001 |
| Body Mass (kg) | 70(22) | 78.00(28.50) | 0.786 |
| Height (cm) | 168.17±7.94 | 165.22±9.02 | 0.666 |
| BMI (kg×m^2^) | 27.4(6) | 28.05(10.80) | 0.266 |
|  |  |  |  |
| Biological Sex |  |  | 0.090 |
| Female | 24 (82.8%) | 35 (94.6%) |  |
| Male | 5 (17.2%) | 1 (2.7%) |  |
|  |  |  |  |
| Employment status |  |  | <0.01* |
| Full-time | 5 (17.2%) | 7 (18.9%) |  |
| Part-time | 4 (13.8%) | 7 (18.9%) |  |
| Not Employed | 6 (20.7%) | 5 (13.5%) |  |
| Retired | 11 (37.9%) | 0 |  |
| Student | 3 (10.3%) | 16 (43.2%) |  |
| Prefer not to say | 0 | 2 (5.4%) |  |
|  |  |  |  |
| Education |  |  | 0.675 |
| Secondary School | 2 (6.9%) | 2 (5.4%) |  |
| Sixth from College | 5 (17.2%) | 8 (21.6%) |  |
| Vocation Qualification | 3 (10.3%) | 2 (5.4%) |  |
| Undergraduate University | 7 (24.1%) | 14 (37.8%) |  |
| Postgraduate | 12 (41.4%) | 11 (29.7%) |  |
|  |  |  |  |
| Marital Status |  |  | 0.041* |
| Married | 12 (41.4%) | 13 (35.1%) |  |
| Cohabiting | 5 (17.2%) | 6 (16.2%) |  |
| Single | 6 (20.7%) | 17 (45.9%) |  |
| Separated | 2 (6.9%) | 0 |  |
| Divorced | 4 (13.8%) | 0 |  |
| Prefer not to say | 0 | 1 (2.7) |  |
|  |  |  | 0.123 |
| Duration of Condition |  |  |  |
| 3-6 months | 0 | 2 (5.4%) |  |
| 6-12 months | 0 | 2 (5.4%) |  |
| 1-2 years | 1 (3.4%) | 5 (13.5%) |  |
| Over 2 years | 28 (96.6%) | 28 (75.7%) |  |
|  |  |  |  |
| Received Pacing Advice |  |  | <0.001* |
| Yes | 18 (62.1%) | 7 (18.9%) |  |
| No | 11 (37.9%) | 30 (81.1%) |  |
|  |  |  |  |
| Length of Time Pacing Advice Received (For the group that only received fatigue management advice n=25) |  |  | 0.005* |
| 1-6 months | 5 (27.6%) | 4 (57.14%) |  |
| 6-12 months | 2 (11.2%) | 1 (14.29%) |  |
| 1-5 years | 9 (50.0%) | 2 (28.57%) |  |
| 5-10 years | 2 (11.2%) | 0 |  |
|  |  |  |  |
| Fatigue | 6.11(2.28) | 5.77(1.84) | 0.660 |
| Engagement in Activity Pacing | 17.69±5.47 | 17.65±6.16 | 0.368 |
| Perceived Risk of Overactivity | 8(2) | 7.50(3) | 0.609 |
| Self-regulation of PA | 33.69±9.09 | 29.28±11.47 | 0.193 |
| Self-reported PA | 2079.00(3725.00) | 2589.00(4563.75) | 0.452 |
| Overall HRQoL | 56.46±16.14 | 53.91±19.39 | 0.209 |
| Physical Well-being | 15.86±6.02 | 13.46±7.26 | 0.383 |
| Social Well-being | 14.90±5.88 | 15.40±5.87 | 0.877 |
| Emotional Well-being | 13.76±4.40 | 11.81±5.37 | 0.238 |
| Functional Well-being | 11.93±5.79 | 13.32±6.57 | 0.473 |

Values presented are M±SD for normally distributed variables, Mdn (IQR) for non-normally distributed variables or N (%) for categorial variables; BMI, body mass index; kg, kilograms; cm, centimetres; m^2^, metres squared; HRQoL, health-related quality of life; PA, physical activity
